# Repair of orbital bone defects in canines using grafts of enriched autologous bone marrow stromal cells

**DOI:** 10.1186/1479-5876-12-123

**Published:** 2014-05-11

**Authors:** Yefei Wang, Xiaoping Bi, Huifang Zhou, Yuan Deng, Jing Sun, Caiwen Xiao, Ping Gu, Xianqun Fan

**Affiliations:** 1 Department of Ophthalmology, Ninth People’s Hospital, Shanghai Jiao Tong University School of Medicine, Shanghai, China

## Abstract

**Backgroud:**

Bone tissue engineering is a new approach for the repair of orbital defects. The aim of the present study was to explore the feasibility of tissue-engineered bone constructed using bone marrow stromal cells (BMSCs) that were rapidly isolated and concentrated from bone marrow (BM) by the red cell lysis method, then combined with β-tricalcium phosphate (β-TCP) to create grafts used to restore orbital bone defects in canines.

**Methods:**

In the experimental group, grafts were constructed using BMSCs obtained by red cell lysis from 20 ml bone marrow, combined with β-TCP and BM via the custom-made stem cell-scaffold device, then used to repair 10 mm diameter medial orbital wall bony defects in canines. Results were compared with those in groups grafted with BM/β-TCP or β-TCP alone, or with defects left untreated as controls. The enrichment of BMSCs and nucleated cells (NCs) in the graft was calculated from the number in untreated bone marrow and in suspensions after red cell lysis. Spiral computed tomography (CT) scans were performed 1, 4, 12 and 24 weeks after implantation in all groups. Gross examination, micro-CT and histological measurements were performed 24 weeks after surgery. The results were analyzed to evaluate the efficacy of bone repair.

**Results:**

The number of NCs and of colony-forming units within the scaffolds were increased 54.8 times and 53.4 times, respectively, compared with untreated bone marrow. In the BMSC-BM/β-TCP group, CT examination revealed that the scaffolds were gradually absorbed and the bony defects were restored. Micro-CT and histological examination confirmed that the implantations led to good repair of the defects, with 6 out 8 orbital defects completely restored in the experimental group, while by contrast, the grafts in the control groups did not fully repair the bony defects, a difference which was statistically significant (p < 0.05).

**Conclusions:**

Tissue-engineered bone, constructed using BMSCs isolated by red cell lysis of BM, can restore critical-sized orbital wall defects in canines.

## Introduction

Orbital bone defects can occur as a result of facial trauma, tumor invasion, congenital malformation, or inflammatory disease, and often lead to impairment of visual function and deformity of facial appearance. Such defects usually arise at the inferior and medial orbital walls because of their thickness of only 0.3 to 0.9 mm, and cannot heal spontaneously, since the orbital contents occupy the defect area and herniate into the paranasal sinus through the defect. Consequently orbital bone defects often need proper and precise repair and reconstruction.

Clinical reconstruction of bone defects has been performed using various materials including titanium, porous polyethylene, hydroxyapatite, silicone, and bone grafts. The above artificial materials act as non-absorbable foreign bodies and have been associated with infection and extrusion, as well as cyst formation [1-3]. Although considered as the ‘gold standard’ for bone defect repair, autologous and allogenous bone grafting is limited by certain disadvantages, such as donor site morbidity, pathogen transfer, and long recovery period [4,5]. To overcome these issues, tissue engineering has proven to be a promising approach for the restoration of orbital bone defects [6-11].

Bone tissue engineering combines three key elements: cells with osteogenic potential, osteoconductive scaffolds, and osteoinductive growth factors. The element of the stem or osteoprogenitor cells is the key point of tissue engineering for bone restoration, as they are even capable of accomplishing bone regeneration without scaffolds *in vivo*[12-14]. Compared with other sources of stem cells, bone marrow (BM)-derived mesenchymal stem cells (BMSCs) are the source which has been most frequently applied both clinically and experimentally for bone tissue engineering, since BMSCs have a high osteogenic capability *in vivo* and *in vitro*, and are easy to isolate from aspirated bone marrow that is extensively available. However, BMSCs only comprise approximately 0.001–0.01% of the total population of nucleated cells (NCs) in the bone marrow, which need to be cultivated and expanded *in vitro* for further application. The expansion process *in vitro* involved many problems, such as the time-consuming, high cost, and ethical issues caused by the addition of animal serum, which remain an obstacle toward clinical application of BMSCs. Several methods for concentration of BMSCs have been introduced for bone tissue engineering in the surgical environment. Techniques based on density gradient centrifugation for concentration of bone marrow cells need too much bone marrow (52–252 ml) and special equipment [15-21]. In selective cell retention (SCR) technology, marrow is passed through a porous matrix which utilizes the physical principles of an affinity column to enrich osteoprogenitor cells that are more prone to attach to the matrix than other NCs, while hematopoietic cells pass through. However this method requires scaffolds in granular form with specific volume (1.5-10 ml), and there are no standards for SCR devices or bone marrow flow rate [22-25]. In addition, both these technologies have a low concentration rate for BMSCs (<6 times) leading to a tremendous waste of bone marrow. Isolation of BMSCs by red blood cell lysis is a faster and more efficient method than density gradient centrifugation and has high utilization of bone marrow [26,27], thus it shows promise in clinical applications for bone tissue engineering.

In this study, we constructed tissue-engineered bone using BMSCs obtained by red blood cell lysis of bone marrow combined with porous β-tricalcium phosphate (β-TCP), a degradable bioceramic scaffold that has been extensively used in clinical and experimental bone tissue engineering [9,15,25,28], and used this to restore medial orbital wall defects in canines. The overall objectives of this study were therefore (1) to evaluate the potential of the constructed tissue-engineered bone; (2) to examine the increase in the numbers of NCs and BMSCs infused in the β-TCP; (3) to investigate the effectiveness of this technique in repairing orbital wall defects.

## Materials and methods

### Animals

All experimental procedures were performed following animal protocols approved by the Animal Care and Experiment Committee of Shanghai Jiao Tong University School of Medicine. Animals were housed in the Animal Center of Ninth People's Hospital Shanghai Jiao Tong University School of Medicine. A total of 14 adult beagle dogs in healthy condition, aged 1-year-old with an average weight of 14.5 kg were used in this study.

### Bone marrow aspiration

After induction of general anesthesia with ketamine (10 mg/kg) and 5% pentobarbital (0.5 ml/kg), 20 ml bone marrow was collected into a 50 ml centrifuge tube containing 1 ml heparin solution (1000 units/ml) from both sides of the iliac crest area in 2 ml increments from all animals. This method was to reduce the degree of dilution by peripheral blood and obtain the highest quality bone marrow [29].

### Isolation of BMSCs by red blood cell lysis

Each 20 ml heparinized marrow suspension was divided into 2 equal portions, and each portion was mixed with 30 mL 4°C red blood cell lysis buffer (Qcbio Science & Technologies Co., Ltd., Shanghai, China) in a 50 ml centrifuge tube, and incubated for 10 min at room temperature on a horizontal shaker. The suspensions were then centrifuged at 400 g for 5 min and the supernatant was discarded; the pellet was then washed twice in phosphate buffered saline (PBS).

### Graft preparation

β-TCP scaffolds (Shanghai Bio-Lu Biomaterials Co., Ltd) were molded into 3-mm-high symmetric cylinders 10 mm in diameter as before [30]. With a resistance to pressure of 2–4 Mpa at the circular center, the scaffolds had a volume porosity of 72% with a pore diameter of 450 ± 50 μm, and fully interconnected geometry with interconnection pore size of 120 ± 50 μm.

**Figure 1:**
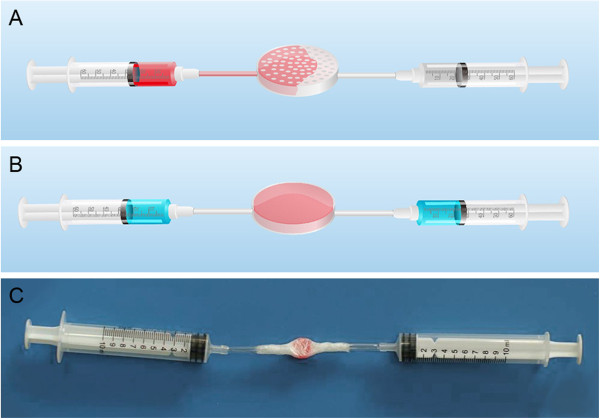
**Illustration of the BMSC/BM and β-TCP combination process using a custom-made stem cell-scaffold combination device. (A)** Conceptual diagram of the combination process. **(B)** Conceptual diagram of the completed process. **(C)** Photograph showing the successful combination of stromal cells with scaffold.

A custom-made cell-scaffold combination device was designed specifically for this study. The device was made from sterilized ophthalmic plastic adhesive membrane (3L Medical Product Group Co., Ltd., Jiangxi, China) encapsulating a β-TCP scaffold centrally and two plastic tubes bilaterally connected with two 10 ml syringes (Figure 1). Before assembly, the β-TCP was hydrated with saline for 2 min, and then the saline was removed. An aliquot of 0.1 ml heparinized marrow suspension was mixed with BMSCs washed by PBS, and then approximately 0.25 ml of the BMSC-BM suspension was fully introduced into the scaffold under low pressure via the device (Figure 1). The BMSC-BM/β-TCP composites were then incubated at 37°C, under conditions of 5% CO_2_, 95% O_2_ and 100% saturation, for 2 h to allow the cells to attach before implantation, while β-TCP alone was hydrated with 0.25 ml saline for 2 h under the same incubation conditions before implantation.

### Surgical procedure

**Figure 2:**
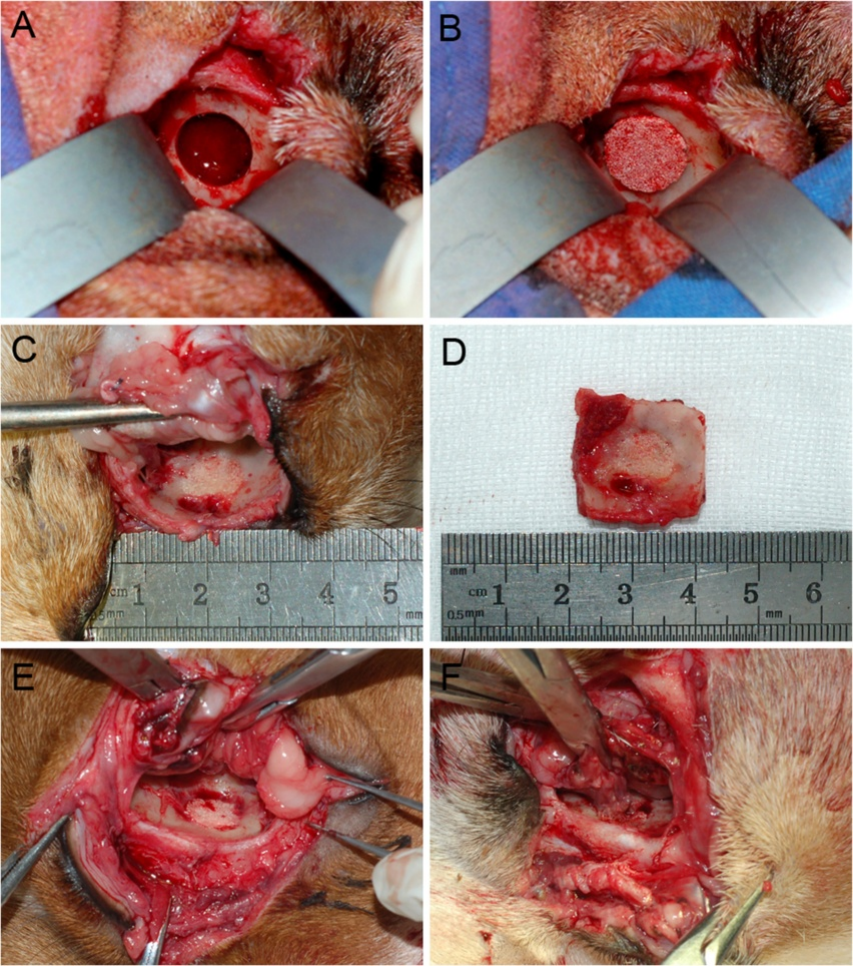
**Repair the medial wall defect and specimen harvest at 24 weeks post-operatively. (A)** 10 mm in diameter defect in the orbital medial wall. **(B)** The graft was implanted into the defect. **(C)** The BMSC-BM/β-TCP group shows bony union achieving around the entire defects. **(D)** Gross view of the specimen. **(E)** The BM/β-TCP group shows new bone formation do not extent to the middle of the grafts, where orbital soft tissue adhered. **(F)** The β-TCP group shows grafts are still visible as porous cylinder shapes, with a raised surface.

The beagles were anesthetized using a combination of ketamine (10 mg/kg) and 5% sodium pentobarbital (0.5 ml/kg) given subcutaneously. The skin around the medio-inferior orbital rim was shaved and disinfected. A crescent-shaped incision was made to access the medial wall, and subcutaneous tissue, muscle and periosteum were separated. A full thickness bony defect with a diameter of 10 mm was created using a 10-mm diameter trephine, leaving at least 5 mm of the orbital anterior rim. The periosteum to a distance of 5 mm around the defect and the nasal sinus mucosal membrane adjacent to the defect were removed. The wound was irrigated with copious quantities of saline to remove any remaining fragments of cancellous bone. Twenty-four medial side orbital bone defects in 12 beagles were randomly divided into four groups: BMSCs-BM/β-TCP composite (n = 8), BM/β-TCP composite (n = 6), β-TCP alone (n = 6), or were left untreated (n = 4), and 2 remaining dogs were used as normal controls (Figure 2A and B). Upon completion of the procedure, the incisions were carefully closed in layers with 4–0 nylon. Post-operatively, 0.8 million units of penicillin were given intramuscularly twice a day for three days.

### Enrichment of NCs and BMSCs in the graft

The BMSC-BM suspensions loaded onto the scaffold were processed by red blood cell lysis then washed twice in PBS, in a process involving three centrifugation steps that resulted in cell loss. Knowing the numbers of NCs and BMSCs in the original BMSC-BM suspensions and the second PBS suspensions, cell loss could be calculated after two centrifugation steps, then the numbers of NCs and BMSCs after three centrifugation steps in the BMSC-BM suspensions could be deduced, and the fold concentration of NCs and BMSCs in the graft compared with the BM could be obtained.

The number of NCs was counted using a hemocytometer (Beckman Coulter HMX); 0.5 ml of the original heparinized marrow suspensions and 0.5 mL of the second PBS suspensions were tested. The number of BMSCs was determined using an established colony forming assay [31], in which the number of colony-forming units (CFUs) expressing alkaline phosphatase was manually counted. Aliquots of 0.2 ml of the original heparinized marrow suspensions and 0.2 ml of the second PBS suspensions were directly added into 6-well plate then mixed with 2 ml α-minimum essential medium (α-MEM, Gibco, Carlsbad, CA) containing 10% fetal bovine serum (Hyclone, Logan, UT), 50 μM ascorbic acid, 10 mM β-glycerophosphate and 10 nM dexamethasone (Sigma, St Louis, MO), respectively. Cells were cultured at 37°C in a humidified atmosphere of 5% CO_2_. The culture medium was exchanged after 48 h and then every 2 days. On day 10, alkaline phosphatase staining was performed *in situ*. Colonies containing 10 or more cells expressing alkaline phosphatase were counted as osteoprogenitor cells.

### Spiral computed tomography (CT) scanning

At 12 and 24 weeks post-operation under general anesthesia, canine head CT scanning was performed using multi-slice spiral CT (GE Lightspeed Ultra 16, Milwaukee, WI), three-dimensional (3D) images were reconstructed.

### Gross observation and micro-CT measurement

All animals were euthanized at 24 weeks postoperatively, and the implants and surrounding tissues were harvested using a saw. Specimens were subjected to micro-CT (μ80, Scanco Medical, Zurich, Switzerland) measurement. 3D images were reconstructed, and the percentage of the defect covered by new bone tissue was calculated.

### Histological analysis

After harvest, the specimens were fixed in 10% formalin solution and dehydrated through a graded series of ethanol, then embedded in methylmethacrylate. The specimens were cut in a longitudinal fashion. The sections were then surface-stained with Van Gieson’s picro-fuchsin for histomorphometric analysis as before [32]. The surface areas of the newly-formed bone (stained red) and the TCP residue (stained black) were measured using Image Pro Plus™ (Media Cybernetics, Silver Springs, MD).

### Statistical analysis

All data are presented as the mean ± SD. Using the software SPSS 17.0 (SPSS Inc., Chicago, IL), Fisher’s exact test was used to detect differences between groups with the level of significance set at *P* < 0.05.

## Results

All animals maintained normal daily activities and remained in healthy condition post-operation. There were no cases of hemorrhage or infection around the incisions, and swelling disappeared within 2 weeks postoperatively.

### Enrichment of NCs and BMSCs

The mean number of NCs in the marrow samples was (13.3 ± 9.0) × 10^6^/ml, with BMSCs present at (128.6 ± 51.1)/ml. After two centrifugation steps, the numbers of NCs and BMSCs were (9.9 ± 7.0) × 10^6^/ml and (93.1 ± 36.9)/ml respectively. The loss rates of NCs and BMSCs per centrifugation step [(14.68 ± 0.03) % and (14.70 ± 0.02)%] were calculated, and the numbers of NCs and BMSCs after the third centrifugation step were (169.3 ± 123.2) × 10^6^ and (1585.6 ± 631.8), which were also the counts of NCs and BMSCs combined in the grafts. Compared with a BM graft, the BMSC-BM graft contained on average 54.8 times more NCs and 53.4 times more BMSCs (Table 1).

**Table 1:** Enrichment of NCs and BMSCs

	**Number of NCs in the BM (×10**^**6**^**/mL)**	**Number of NCs after twice centrifugation (×10**^**6**^**/mL)**	**Number of BMSCs in the BM (/mL)**	**Number of BMSCs after twice centrifugation (/mL)**	**Loss rate of NCs after per centrifugation step**	**Loss rate of BMSCs after per centrifugation step**	**Calculated number of NCs after the third centrifugation step (×10**^**6**^**/mL)**	**Calculated number of BMSCs after the third centrifugation step (/mL)**	**Enrichment of NCs in the grafts**	**Enrichment of BMSCs in the grafts**
Sample 1	20.8	14.2	160	110	17.40%	17.00%	234.6	1826	48.5	49.1
Sample 2	5.6	4.4	110	80	11.40%	14.70%	78	1365	60	53.3
Sample 3	10.4	8.4	85	65	10.10%	12.60%	151	1136	62.9	57.8
Sample 4	10.6	8	115	85	20.30%	14.00%	127.5	1462	52	54.7
Sample 5	7	5	70	55	15.50%	11.40%	84.5	975	51.7	60.3
Sample 6	8.7	6.3	135	90	14.90%	18.40%	107.2	1469	53.5	45
Sample 7	10.4	7.3	120	85	16.20%	15.80%	122.3	1431	50.3	51.7
Sample 8	32.5	25.4	235	175	11.60%	13.70%	449.1	3021	59.6	55.4
Average	**13.3**	**9.9**	**128.8**	**93.1**	**14.68%**	**14.70%**	**169.3**	**1585.6**	**54.8**	**53.4**

### Spiral CT scanning

**Figure 3:**
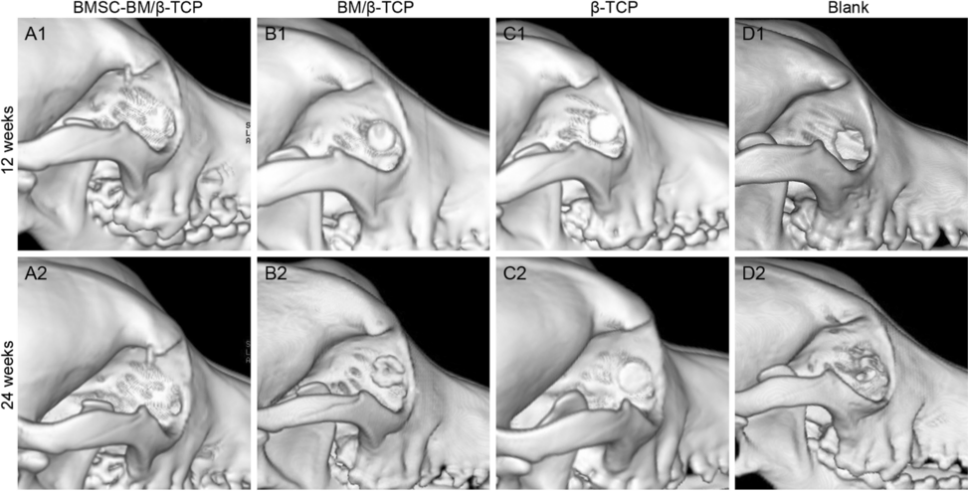
**Three-dimensional CT image analysis of the repaired orbital bone defects in the various group at 12 and 24 weeks. (A1-2)** In the BMSC-BM/β-TCP group, the graft gradually absorbed and accompanied with new bone formation that is identical to normal orbits. **(B1-2)** In the BM/β-TCP group, the graft absorbed with less bone regeneration. **(C1-2)** In the β-TCP group, the scaffold remained original shape. **(D1-2)** No obvious new bone formation in the blank group.

Twelve weeks after implantation, the edges of the BMSC-BM/β-TCP composites appeared faint, and cortical bone replaced the grafts. BM/β-TCP and β-TCP grafts retained their original shape, and little cortical bone could be seen ingrown at the periphery. Twenty-four weeks after surgery, in the BMSC-BM/β-TCP group, graft thickness was identical to that of a normal orbit, and grafts were replaced by new cortical bone, which had assumed the contour of a normal orbit. In the BM/β-TCP group, ossifications had developed around the rim of the implant, but the shape of the graft was still identifiable. There was no obvious new bone formation around grafts in the β-TCP group, and contour changes of the graft were minimal. In the untreated group, no callus formation was observed during the entire post-operative period (Figure 3).

### Gross view

After harvesting the specimens, the BMSC-BM/β-TCP group showed new bone formation filling the defects, with bony union being achieved all around the defects, and the surface of the new bone level with adjacent bone and appearing whitish in color (Figure 2C and D). In the BM/β-TCP group, new bone formation was observed around the defects, but this did not extend to the middle of the grafts, where orbital soft tissue adhered (Figure 2E). In the β-TCP group, grafts were still visible as porous cylinder shapes, with a raised surface compared to that of the adjacent orbit (Figure 2F).

### Micro-CT measurement

**Figure 4:**
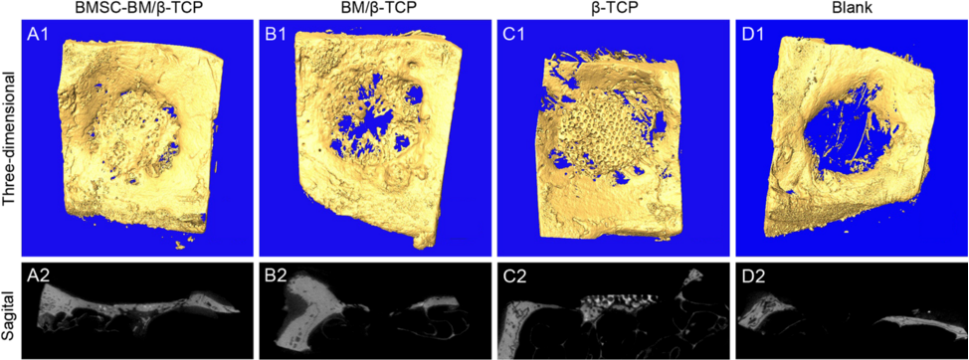
**Micro-CT imaging analysis at 24 weeks post-operatively.** The top and bottom panels represent the 3D-images from the face to the eye ball and the images of section view.

Micro-CT images were taken at 24 weeks post-operation (Figure 4). New bone regeneration across the diameter of the defects occurred in 6 out of 8 BMSC-BM/β-TCP composites, with > 90% of the defect filled with newly-formed bone tissue and smoothly remodeled bone contours. The new bone and the bony defect were connected by a synostosis, with an indistinct boundary, and a few residual β-TCP particles were scattered among the newly formed bone in transection view. Only one of the BM/β-TCP composites exhibited new bone regeneration extending the total length of the defect, but all grafts restored < 50% of the defect. In transection plane, new bone formation was observed peripherally, but the center of the scaffolds was completely degraded. In the TCP group, bone union occurred here and there around the defect, the non-degraded scaffold retained its original shape, and only a small amount of new bone was formed under the non-degraded scaffold. In the untreated group, fibrous tissue and mucosal membrane occupied the defects, no obvious new bone formation was detected.

### Histological analysis

**Figure 5:**
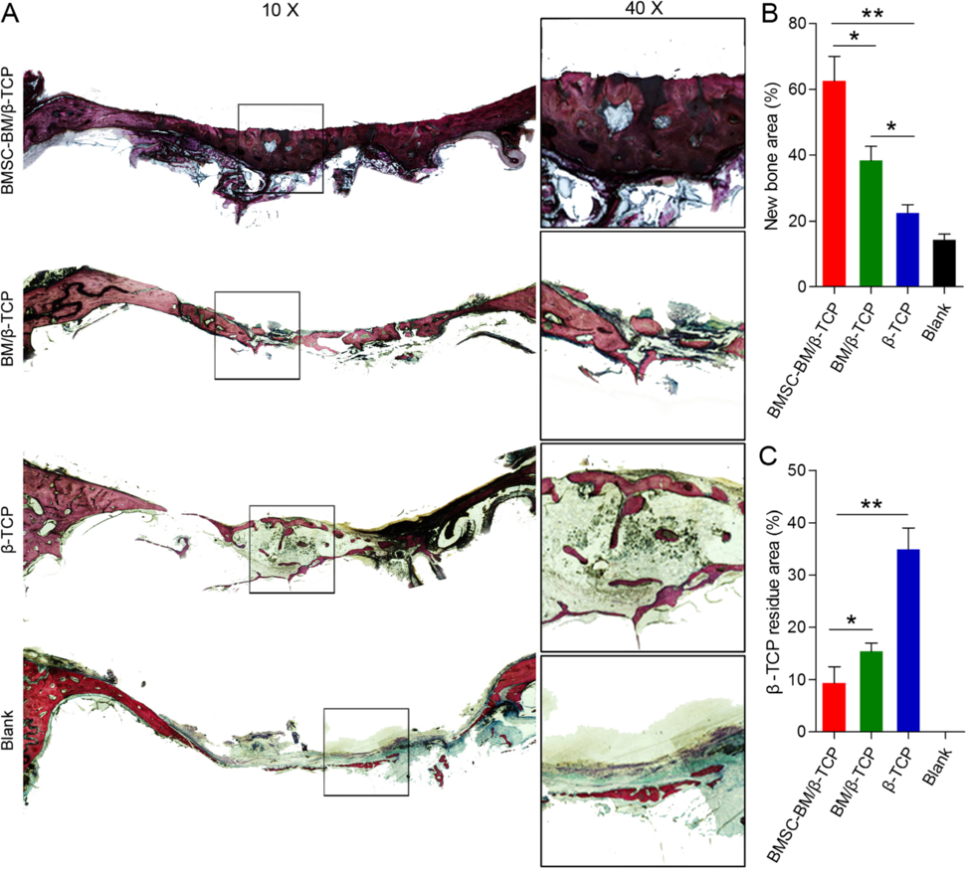
**Van Gieson’s picro-fuchsin staining of the samples for histological analysis of new bone formation and β-TCP residue at 24 weeks post-operatively. (A)** From top to bottom: BMSC-BM/β-TCP, BM/β-TCP, β-TCP and blank group (original magnification 10× and 40×). New bone appears red, and β-TCP appears black. **(B, C)** There were significant differences between the BMSC-BM/β-TCP group and control groups in the new bone area and β-TCP residue (*p < 0.05, **p < 0.01).

Histology of hard tissue sections using Stevenel’s blue/ Van Gieson’s picro-fuchsin staining at 24 weeks post-operation was performed (Figure 5). Newly-formed bone tissue was stained red and had a woven, trabecular appearance with complete bone union in BMSC-BM/β-TCP grafts, which differed from the lamellar bones of the normal group. In the BM/β-TCP group, the new bone was intermittent, porous and thin. There was no obvious bone union, only a small amount of new bone formation, and much scaffold remaining in the TCP group. In the untreated group, only a trivial amount of new bone was formed at the interface between the orbital contents and the sinus membrane. The percentage of new bone area in the BMSC-BM/β-TCP group (62.57 ± 7.40%) was obviously and significantly greater than that in the control groups (38.37 ± 4.27% in the BM/β-TCP group; 22.39 ± 2.56% in the β-TCP group; and 14.23 ± 1.85% in the untreated group; p *< 0.05*). Less β-TCP residue remained in the BMSC-BM/β-TCP group (9.36 ± 3.12%) compared to the other groups (15.33 ± 1.61%, in the BM/β-TCP group; 34.92 ± 4.09% in the β-TCP group; p < 0.05).

## Discussion

Various strategies of enriching stem/progenitor cells for use in cell-based bone regeneration without expansion *in vitro* have been explored both clinically and experimentally with varying degrees of success. Connolly demonstrated that bone marrow concentrated by unit gravity sedimentation or centrifugation could significantly increase deposition of calcium in ectopic sites of the rabbit, which allowed four to five times as many NCs to be implanted [33]. A cell separator based on differences in cell density in bone marrow, used the centrifugation method to concentrate progenitor cells and mononuclear cells, and has been applied in several clinical studies of bone regeneration [13-15,19]. Studies using this cell separator revealed a positive correlation between new bone regeneration and the number and concentration of osteoprogenitors in the bone marrow graft. Use of the cell separator reduced 150 ml and 300 ml of bone marrow to 30 ml and 50 ml of mononuclear cell suspension and concentrated the BMSCs (CFU-F) approximately 4.3 and 4.2 times, respectively [13,14,19]. In a study, good results were achieved in 95.1% of cases of posterior spinal fusion with grafts constructed of β-TCP and bone marrow stem cells enriched by the separator, which reduced 252 ml of marrow to 45 ml of enriched stem cell suspension and concentrated the BMSCs 4.3 times [15]. Recently, bone marrow mononuclear cells concentrated by a novel point-of-care bone marrow aspirate concentrate (BMAC) system, which relies on density gradient centrifugation, was shown to enhance bone regeneration and healing *in vivo*[16-18,20,21]. In comparison with the Ficoll isolation procedure, the BMAC method yielded a 2.4-fold higher total number of nucleated cells and a similar number of BMSCs [17,21].

The selective cell retention method that is based on BMSCs property of adhesion to a matrix was used to construct graft materials with enriched osteoprogenitor cells. BMSCs could be enriched 3.4-5.6 times by SCR method and were more likely to be retained in demineralized cancellous bone matrix than other bone marrow-derived cells, with a selection ratio of 1.9-3.0 [22,24]. Grafts prepared using SCR technology combined with demineralized bone and cancellous chips to repair canine critical-size femoral defects and obtained the same effect as with autologous bone. In their study the osteoprogenitor cells were concentrated 3.6 times and the nucleated cells 1.4 times [23]. This enrichment method to achieve a maximum of 3 times more BMSCs which they combined with β-TCP to achieve similar fusion rates with autografts in an ovine spine fusion model [25].

Although these methods generated the expected results in new bone regeneration, they are not applicable to the repair of orbital wall bone defects. The orbital wall is so thin and lacking in support that it requires thin and rigid scaffolds that possess rather a small volume in total to repair, and as a result the volume of BM needed for the centrifugation method is too large and would result in a great deal of waste, while using SCR enrichment technology for such regular and thin scaffolds is difficult and inefficient. In addition, the specific and expensive equipment needed for the centrifugation technique, and the lack of any standard for the SCR device or the bone marrow flow rate has limited their further application. The red cell lysis method for isolation of BMSCs from BM has proven that it is efficient, faster and more easily standardized for clinical applications of BMSCs [26,27], although, it has never previously been used for tissue-engineered bone reconstruction. Based on the above findings, we used the red cell lysis method to collect NCs from BM and combine them with β-TCP and BM to restore medial orbital wall defects in a canine model in this study.

Lysis with ammonium chloride depends on the continuous flow of NH_3_ and CO_2_ through the cell membrane into erythrocytes, which contain large quantities of carbonic anhydrase that converts NH_3_ to NH_4_^+^ and CO_2_ to HCO_3_^−^. This lysis procedure has a great effect on red blood cells but very few toxic effects on NCs in the BM, such as BMSCs, osteocytes, endothelial cells, reticular cells, or fibroblasts as well as platelets. All these components in 20 ml BM were collected after centrifugation three times and used to construct tissue-engineered bone, which contained 169.3 × 10^6^ BMSCs and 1585.6 NCs. Due to the thin scaffold used in our study, the total volume of the scaffold was very small and less than 0.25 ml, so that NCs were concentrated 54.8 times and BMSCs, 53.4 times. The concentration rates of NCs and BMSCs were quite close and different from those of the SCR method that had a higher concentration rate of BMSCs. In spite of that, the concentration rates of both NCs and BMSCs in our study were much higher, being at least 10 times more compared to those obtained using centrifugation and SCR methods.

The optimal density of stem cells infused in a scaffold is as yet unknown. It could be affected by scaffold properties, sites of transplantation, growth factors and especially cell conditions, including cultivation and expansion *in vitro*, genetic modification, and direct harvest. There are large variations in cell quantities infused into scaffolds when stem cells are expanded *in vitro* or directly concentrated. Zhou [9] and Yuan [28] both transplanted culture-expanded BMSCs with a concentration of 20 × 10^6^ /ml into β-TCP and obtained expected results. Haynesworth considered that the optimal concentration of stem cells infused into scaffolds would range from 0.7 to 20 × 10^6^/ml [34]. Consequently, numbers of culture-expanded stem cells may be thousands of times higher than those obtained by concentration methods or the SCR method of harvest, and hundreds of times higher than those obtained by the red cell lysis method used herein, that would be the closest to the culture-expanded group among the above non-culture methods. In view of the results of previous investigations of orbital bone defects restored using tissue engineering techniques, grafts combined with BM [6], nanocomposites [8] or bone morphogenetic protein-2 (BMP-2) [7] with different kinds of scaffolds, did not lead to complete restoration of orbital bone defects. Hence, since the incorporation of more BMSCs or osteoprogenitor cells could be more beneficial to orbital bone regeneration, we concentrated and combined the highest concentration of BMSCs achieved so far under intro-operative conditions, and succeeded in achieving almost complete repair of the orbital bony defects.

Since it was believed that native BMSCs and β-TCP alone would not be enough to regenerate new orbital bone, a small amount of BM (0.1 ml) was added into the grafts, which provided platelets and plasma to reproduce the native bone marrow environment. In addition, a considerable number of platelets remained in the pellet after red cell lysis, which could release platelet-derived growth factor (PDGF) that both contributes to osteoblast differentiation and helps to stabilize newly-forming vessels, and has been deemed to be a powerful agent for new bone formation [35]. In the experimental group newly-formed orbital bone was also remodeled in response to the orbital stresses at the transplantation site, which comprised the outward pressure of orbital contents and inward pressure of nasal sinus. The orbital contents that mainly consists of eyeball and orbital fat, but paranasal sinus is a bony cavity, the outward pressure is prominent, which made newly-formed orbital bone slightly curved to nasal cavity. As a result, the newly-formed orbital bone exhibited similar bony contour and structures to normal orbital bone.

## Conclusion

In conclusion, The BMSC-BM/β-TCP composites were able to efficiently restore the orbital bone defects, and the macro- and micro-structure of the newly formed bone was quite similar to normal orbital bone, also the degradation of β-TCP scaffold is consistency with the new bone formation. This study revealed that tissue-engineered bone can be constructed using the red cell lysis method under intraoperative conditions. BMSCs and NCs infused into scaffolds were more highly concentrated than by any other method due to the thin structure of the orbital wall and the red cell lysis method used herein, which provided an excellent model for intraoperative bone tissue engineering research.

## Abbreviations

BM: Bone marrow; BMSCs: Bone marrow stromal cells; β-TCP: β-tricalcium phosphate; CT: Spiral computed tomography; NCs: nucleated cells; SCR: Selective cell retention; PBS: Phosphate buffered saline; CFUs: Colony-forming units; α-MEM: α-minimum essential medium; 3D: Three dimensional; BMAC: Bone marrow aspirate concentrate; BMP-2: Bone morphogenetic protein-2; PDGF: Platelet-derived growth factor.

## Competing interests

The authors declare that they have no competing interests.

## Authors’ contributions

YW and XB: performed the experiments in vitro and in vivo, acquisition of data, statistical analysis and drafted the manuscript. HZ and YD: carried out CT and micro-CT analysis. JS and CX: fabricate the β-TCP scaffolds. PG: performed the histological analysis. XF: Participated in research design, the performance of the research, data analysis and the writing of the paper. All authors have read and approved the final manuscript.

## References

[B1] Karsloglu S Serin D Simsek I Ziylan S Implant infection in porous orbital implants Ophthal Plast Reconstr Surg 2006 22 461 466 10.1097/01.iop.0000248156.41020.94 17117103

[B2] Jacono AA Moskowitz B Alloplastic implants for orbital wall reconstruction Facial Plast Surg 2000 16 63 68 10.1055/s-2000-7327 11802348

[B3] Hillier RJ Osborne SF Leatherbarrow B Epithelial inclusion cyst associated with a porous polyethylene orbital floor implant Ophthal Plast Reconstr Surg 2009 25 238 239 10.1097/IOP.0b013e3181a394e7 19454943

[B4] De Long WG Jr Einhorn TA Koval K McKee M Smith W Sanders R Watson T Bone grafts and bone graft substitutes in orthopaedic trauma surgery. A critical analysis J Bone Joint Surg Am 2007 89 649 658 10.2106/JBJS.F.00465 17332116

[B5] Parikh SN Bone graft substitutes: past, present, future J Postgrad Med 2002 48 142 148 12215702

[B6] Rohner D Hutmacher DW Cheng TK Oberholzer M Hammer B In vivo efficacy of bone-marrow-coated polycaprolactone scaffolds for the reconstruction of orbital defects in the pig J Biomed Mater Res B Appl Biomater 2003 66 574 580 12861610 10.1002/jbm.b.10037

[B7] Betz MW Caccamese JF Coletti DP Sauk JJ Fisher JP Tissue response and orbital floor regeneration using cyclic acetal hydrogels J Biomed Mater Res A 2009 90 819 829 18615468 10.1002/jbm.a.32131

[B8] Patel M Betz MW Geibel E Patel KJ Caccamese JF Coletti DP Sauk JJ Fisher JP Cyclic acetal hydroxyapatite nanocomposites for orbital bone regeneration Tissue Eng Part A 2010 16 55 65 19614544 10.1089/ten.TEA.2009.0027

[B9] Zhou H Xiao C Wang Y Bi X Ge S Fan X In Vivo efficacy of bone marrow stromal cells coated with beta-tricalcium phosphate for the reconstruction of orbital defects in canines Invest Ophthalmol Vis Sci 2011 52 1735 1741 10.1167/iovs.10-5988 21087968

[B10] Xiao C Zhou H Ge S Tang T Hou H Luo M Fan X Repair of orbital wall defects using biocoral scaffolds combined with bone marrow stem cells enhanced by human bone morphogenetic protein-2 in a canine model Int J Mol Med 2010 26 517 525 20818491 10.3892/ijmm_00000494

[B11] Xiao C Zhou H Liu G Zhang P Fu Y Gu P Hou H Tang T Fan X Bone marrow stromal cells with a combined expression of BMP-2 and VEGF-165 enhanced bone regeneration Biomed Mater 2011 6 015013 10.1088/1748-6041/6/1/015013 21252414

[B12] Connolly JF Guse R Tiedeman J Dehne R Autologous marrow injection as a substitute for operative grafting of tibial nonunions Clin Orthop Relat Res 1991 259 270 2019059

[B13] Hernigou P Beaujean F Treatment of osteonecrosis with autologous bone marrow grafting Clin Orthop Relat Res 2002 14 23 12461352 10.1097/00003086-200212000-00003

[B14] Hernigou P Poignard A Beaujean F Rouard H Percutaneous autologous bone-marrow grafting for nonunions. Influence of the number and concentration of progenitor cells J Bone Joint Surg Am 2005 87 1430 1437 10.2106/JBJS.D.02215 15995108

[B15] Gan Y Dai K Zhang P Tang T Zhu Z Lu J The clinical use of enriched bone marrow stem cells combined with porous beta-tricalcium phosphate in posterior spinal fusion Biomaterials 2008 29 3973 3982 10.1016/j.biomaterials.2008.06.026 18639333

[B16] Wongchuensoontorn C Liebehenschel N Schwarz U Schmelzeisen R Gutwald R Ellis E 3rd Sauerbier S Application of a new chair-side method for the harvest of mesenchymal stem cells in a patient with nonunion of a fracture of the atrophic mandible–a case report J Craniomaxillofac Surg 2009 37 155 161 10.1016/j.jcms.2008.11.002 19155179

[B17] Sauerbier S Stricker A Kuschnierz J Bühler F Oshima T Xavier SP Schmelzeisen R Gutwald R In vivo comparison of hard tissue regeneration with human mesenchymal stem cells processed with either the FICOLL method or the BMAC method Tissue Eng Part C Methods 2010 16 215 223 10.1089/ten.tec.2009.0269 19473102

[B18] Sauerbier S Rickert D Gutwald R Nagursky H Oshima T Xavier SP Christmann J Kurz P Menne D Vissink A Bone marrow concentrate and bovine bone mineral for sinus floor augmentation: a controlled, randomized, single-blinded clinical and histological trial–per-protocol analysis Tissue Eng Part A 2011 17 2187 2197 10.1089/ten.tea.2010.0516 21529247

[B19] Hernigou P Daltro G Filippini P Mukasa MM Manicom O Percutaneous implantation of autologous bone marrow osteoprogenitor cells as treatment of bone avascular necrosis related to sickle cell disease Open Orthop J 2008 2 62 65 10.2174/1874325000802010062 19478932 PMC2687112

[B20] Hendrich C Franz E Waertel G Krebs R Jager M Safety of autologous bone marrow aspiration concentrate transplantation: initial experiences in 101 patients Orthop Rev (Pavia) 2009 1 e32 21808691 10.4081/or.2009.e32 PMC3143993

[B21] Hermann PC Huber SL Herrler T von Hesler C Andrassy J Kevy SV Jacobson MS Heeschen C Concentration of bone marrow total nucleated cells by a point-of-care device provides a high yield and preserves their functional activity Cell Transplant 2008 16 1059 1069 18351022

[B22] Muschler GF Matsukura Y Nitto H Boehm CA Valdevit AD Kambic HE Davros WJ Easley KA Powell KA Selective retention of bone marrow-derived cells to enhance spinal fusion Clin Orthop Relat Res 2005 242 251 15738828 10.1097/01.blo.0000149812.32857.8b PMC1425153

[B23] Brodke D Pedrozo HA Kapur TA Attawia M Kraus KH Holy CE Kadiyala S Bruder SP Bone grafts prepared with selective cell retention technology heal canine segmental defects as effectively as autograft J Orthop Res 2006 24 857 866 10.1002/jor.20094 16602110

[B24] Muschler GF Nitto H Matsukura Y Boehm C Valdevit A Kambic H Davros W Powell K Easley K Spine fusion using cell matrix composites enriched in bone marrow-derived cells Clin Orthop Relat Res 2003 102 118 12567137 10.1097/00003086-200302000-00018 PMC1425047

[B25] Gupta MC Theerajunyaporn T Maitra S Schmidt MB Holy CE Kadiyala S Bruder SP Efficacy of mesenchymal stem cell enriched grafts in an ovine posterolateral lumbar spine model Spine 2007 32 720 726 10.1097/01.brs.0000258863.40984.32 17414903

[B26] Horn P Bork S Diehlmann A Walenda T Eckstein V Ho AD Wagner W Isolation of human mesenchymal stromal cells is more efficient by red blood cell lysis Cytotherapy 2008 10 676 685 10.1080/14653240802398845 18985474

[B27] Horn P Bork S Wagner W Standardized isolation of human mesenchymal stromal cells with red blood cell lysis Methods Mol Biol 2011 698 23 35 10.1007/978-1-60761-999-4_3 21431508

[B28] Yuan J Cui L Zhang WJ Liu W Cao Y Repair of canine mandibular bone defects with bone marrow stromal cells and porous beta-tricalcium phosphate Biomaterials 2007 28 1005 1013 10.1016/j.biomaterials.2006.10.015 17092556

[B29] Muschler GF Boehm C Easley K Aspiration to obtain osteoblast progenitor cells from human bone marrow: the influence of aspiration volume J Bone Joint Surg Am 1997 79 1699 1709 9384430 10.2106/00004623-199711000-00012

[B30] Zhou H Deng Y Bi X Xiao C Wang Y Sun J Gu P Fan X Orbital wall repair in canines with beta-tricalcium phosphate and induced bone marrow stromal cells J Biomed Mater Res B Appl Biomater 2013 101B 1340 1349 23687075 10.1002/jbm.b.32951

[B31] Majors AK Boehm CA Nitto H Midura RJ Muschler GF Characterization of human bone marrow stromal cells with respect to osteoblastic differentiation J Orthop Res 1997 15 546 557 10.1002/jor.1100150410 9379264

[B32] Deng Y Zhou H Zou D Xie Q Bi X Gu P Fan The role of miR-31-modified adipose tissue-derived stem cells in repairing rat critical-sized calvarial defects Biomaterials 2013 34 6717 6728 10.1016/j.biomaterials.2013.05.042 23768901

[B33] Connolly J Guse R Lippiello L Dehne R Development of an osteogenic bone-marrow preparation J Bone Joint Surg Am 1989 71 684 691 2732257

[B34] Haynesworth SE Goshima J Goldberg VM Caplan AI Characterization of cells with osteogenic potential from human marrow Bone 1992 13 81 88 10.1016/8756-3282(92)90364-3 1581112

[B35] Caplan AI Correa D PDGF in bone formation and regeneration: new insights into a novel mechanism involving MSCs J Orthop Res 2011 29 1795 1803 10.1002/jor.21462 21618276

